# Validation of Content for an App for Caregivers of Stroke Patients through the Delphi Method

**DOI:** 10.3390/ijerph19127523

**Published:** 2022-06-20

**Authors:** Ismael Andrades-González, Jesús Molina-Mula

**Affiliations:** 1 Son Espases University Hospital, Ctra Valldemossa, 79, 07120 Palma, Spain; 2 Nursing and Physiotherapy Department, University of Balearics Island, 07122 Palma, Spain; jesus.molina@uib.es

**Keywords:** nursing, caregivers, Delphi method, stroke, e-health, consensus, validity, expert judgment, methodology

## Abstract

The aim of this study was to reach a consensus among experts (using the Delphi technique) to validate the informative content that should be included in an App to be used by informal caregivers of stroke patients in order to improve their quality of life, facilitating access to the health care system and involving them in their own health. This technique was developed between June and December 2021. The group of experts was selected on the basis of previously established criteria, and the coefficient of variation (v) was used as a measure of consensus. In addition, the concordance index was calculated to determine the stability of the different rounds. In the first round, the preliminary content, previously elaborated by the research group, was evaluated as very appropriate for the objectives set (N-P < 1.07). In addition, averages of 4.5 out of five and a coefficient of variation of less than 0.5 were obtained, confirming the consensus. In the second round, suggestions were made by the experts on how to improve the content of the information, obtaining 100% agreement with the results obtained in the first round. The results obtained allow a positive evaluation of the use of the Delphi method for the elaboration of the information to be housed in an App.

## 1. Introduction

It has been widely reported that the number of strokes will rise by 27% in the next 25 years (Spanish Society of Neurology (SEN), s. f. [1]). It is a disease that affects approximately 15 million people worldwide each year, with 30% of those affected dying and two-thirds of survivors suffering from disability aftereffects (Tejada Meza et al., 2019) [2]. When there is a health problem, there is usually a loss or reduction in quality of life. Meanwhile, this alteration in life quality sometimes lasts a long time and affects other people in the patient’s environment. This is the situation of informal caregivers for stroke patients (Bierhals et al., 2019) [3].

Quality of life is a broad concept that encompasses a multitude of disciplines; to better analyze it, health-related quality of life (HRQoL) has emerged, which relates individuals’ perceptions of health, including objective and subjective aspects (Caqueo-Urízar A and Urzúa M., 2012) [4]. Although there is no single definition of life quality, it is comprised of several common dimensions, such as life satisfaction, subjective well-being, health status, mental health, and happiness (Andrades-González et al., 2021) [5]. The aftereffects of a stroke cause dependency in daily activities that must be provided by those around them (usually family members). These people’s quality of life deteriorates over time, affecting their physical, mental, and social health (Woodford J et al., 2017) [6].

According to a recent systematic review with meta-analysis, there is evidence that an e-Health-based intervention has positive effects on depressive symptoms, caregiver use of medical care, caregiver burden, and physical health (Andrades-González et al., 2021) [5]. In addition, during the intervention, the majority of the interventions examined were dominated by leaflet guidance and counseling, so educational interventions reflected a positive impact on caregiver burden.

In order to design and develop an App for stroke caregivers, it is essential to introduce a section about the disease’s impact, risk factors, and sequelae. It must also include the changes that the caregiver will experience due to caring for a dependent person. Moreover, it requires advice in the form of video tutorials and support material, as well as informal caregiver associations that have proven to be effective (Andrades-González et al., 2021 [5]).

The research team of this project set out to create an App aimed at caregivers of stroke patients to improve their quality of life, and that would contain information about the disease, as well as content that will help to improve the day to day life as a caregiver. In addition, they would have a forum to communicate and help each other and a chat that would allow them to communicate directly with the health system.

Effectual techniques must be employed to elaborate the information contained in any App. Many studies have shown that the Delphi technique is a practical methodology for obtaining consensus on a phenomenon from expert opinions. Expert opinions are gathered anonymously and in isolation through a multi-stage survey. This method is based on the idea that the consensus of many experts on a subject is more reliable than the individual opinions of the same experts (Hernández-García and Robaina Castillo, 2017) [7].

Delphi, a well-known method in health research, has been used to elicit opinions, identify priorities, and make decisions. Examples of applications in the field of health sciences can be seen in a variety of phenomena; this technique has been used to describe typical and relevant aspects of patients with spondylitis (Boonen et al., 2009) [8], develop scales for the assessment of fatigue in patients with rheumatoid arthritis (Nikolaus et al., 2012) [9] or, in the area of public health, to manage and prevent food-borne diseases (Palomino-Camargo et al., 2018) [10]. In addition, several studies have recently been published to develop consensus recommendations for managing COVID-19 (Martínez-Ezquerro et al., 2021) [11].

To address this issue, a Smartphone App is being developed with the intention of improving the life quality of stroke caregivers. This intervention is supported by studies that have developed e-Health-based interventions for this phenomenon (Andrades-González et al., 2021) [5].

Therefore, the aim of the study is to reach a general consensus among experts on the supportive information to be housed in an App for stroke caregivers.

## 2. Materials and Methods

To reach a general expert consensus on supportive information for caregivers of stroke patients hosted on an App.

For this study, a prospective method was used through the Delphi technique, with the goal of obtaining both quantitative and qualitative information that will help to achieve the objective set out in a consensual manner. Between June and December of 2021, this technique was developed. In order to accomplish this, a group of experts is commonly selected (based on inclusion and exclusion criteria) and given a questionnaire covering various aspects of a given topic. The experts provide their opinions on the subject, which are then anonymously incorporated into the questionnaire (as feedback) and returned to them. This procedure is repeated until the established criteria are met. Once the required rounds are completed and the process is finished, the researcher can interpret the data and draw conclusions (Hernández-García and Robaina Castillo, 2017) [7].

A table of contents was designed first, and then the information to be agreed on for the purpose of the study was created; to this end, a literature search was conducted for clinical practice guidelines and documents from institutions specializing in stroke and informal caregivers (Ferré i Grau et al., 2011; Altuna Azkargota et al., 2018; García Antón et al., 2013; Sierra Díaz and Bravo Piqueras, 2021) [12,13,14,15]. In the next step, a participatory consensus methodology was applied among the researchers involved in the project through an analysis of the literature and brainstorming. After creating and developing the table of contents, all of the content was reviewed and validated by a panel of subject matter experts using the Delphi method.

### 2.1. Sample/Participants

The following criteria were considered to form the expert panel: (1) hold a degree in nursing, medicine, physiotherapy, or social education; (2) have specialized training and/or five years or more experience in stroke and its environment; and (3) have a competence coefficient between 0.5 and 1 (0.5 < k < 1).

The population, from which the expert group (N) was formed consisted of Healthcare professionals in stroke-related care practice who met selection criteria 1 and 2. They were invited to join the expert group via e-mail, where the purpose of the study was explained, and informed consent for their participation was requested.

Although some authors (García and Fernández, 2008) [16] recommend a number of experts ranging from 7 to 30, others, such as Cabero and Barroso (Almenara and Osuna, 2013) [17], argue that the number of experts for the Delphi method cannot be predetermined in a range. The reason can be linked with the point that on many occasions, access to experts with sufficient knowledge of the subject to be addressed is not possible, or the technique must be carried out quickly. For the development of this study, 16 potential participants were contacted, with 13 agreeing to take part.

The competence coefficient (K) was used to assess expert competence (Hernández-García and Robaina Castillo, 2017) [7]. It is commonly calculated by the formula K = (Kc + Ka)/2, where Kc is the knowledge coefficient, and Ka is the argumentation coefficient. To obtain Kc, participants are usually asked to rate their knowledge of the topic under discussion on a scale of 1 to 10. Following that, the expert group members self-assess the degree of influence of their knowledge sources, classifying them as high, medium, or low. Finally, Ka is obtained in this manner, and the K coefficient can be calculated.

### 2.2. Data Collection

The content of the information developed was presented to the experts in the form of 6 sections, and they were asked to rate the relevance of the content in relation to the project objectives using a Likert scale ranging from 1 to 5. On the other hand, they were asked to make suggestions for improving the content. The following indicators were used to investigate the aspects provided: very adequate (5); quite adequate (4); adequate (3); not very adequate (2); and inadequate (1).

The second round was held to reach a consensus on content improvements based on the opinions indicated by the experts in the previous round. The statistical data (percentages, averages, and standard deviations) derived from the first round responses were described in the second round. In addition, improvements to the content based on the suggestions of the first round participants were included, and respondents were asked whether they agreed with the other recommendations.

### 2.3. Data Analysis

After completing the first round of information evaluation, an analysis of the data obtained was conducted, and statistical processing of the results was carried out to demonstrate reliability and test the level of agreement among the participants. In addition, a qualitative analysis was performed to incorporate the improvements and suggestions made by the group of experts. The coefficient of variation (v) was used as a measure of consensus, which indicates the existing dispersion between the assessments. Notably, if the value is equal to or less than 0.5, the difference is assumed to be significant.

### 2.4. Validity and Reliability/Rigour

As a methodological rigor strategy, we used the Concordance Index. The concordance index was calculated after analyzing the second round, which establishes the laps’ stability. This coefficient in the Delphi technique defines disagreement as the existence of a discrepancy between the scores of one round and the other without taking into account the magnitude of the difference. The concordance was found to be significant in this case, and the expert group consultation was thus terminated.

### 2.5. Ethical Considerations

The study was conducted in accordance with the Declaration of Helsinki, and approved by the Ethics Committee of the Balearic Islands (protocol code 4364/20, 24 February 2021) for studies involving humans.

## 3. Results

Table 1 displays the stroke categories and content developed by the researchers involved in the project. The informative and descriptive parts of what the disease is and what it will mean for the caregiver, as well as advice on how to cope with it better, were undertaken under the categories of disease (stroke), advice, and caregivers. In addition, the possibility of creating videos, in which caregivers could be helped in a more graphic way, was introduced. Finally, the associations and resources categories covered the part of existing aid for more information and support.

### 3.1. Group of Experts

Sixteen professionals who met the selection criteria 1 and 2 were contacted to form the expert group. Of these 16, 13 indicated a willingness to participate in the consultation. The participants were 76% (*n* = 10) female and had an average of 13.6 years of experience (Table 2). Additionally, the experts had a medium-high competence coefficient (Table 3; 15% (*n* = 2) showed a high competence level, and 85% (*n* = 11) represented a medium level.

The participating experts were thought to have sufficient expertise to validate and improve the App’s informative content on stroke caregivers.

### 3.2. Development of the Delphi Technique

In the first round, the content developed based on Table 1 was submitted and validated through expert consultation by comparing the N-P values (N = sum of the sum by aspects and P = average by aspects) obtained for each category with the values of the cut-off points (Table 4).

As can be seen in Table 4, the values obtained for each category consulted are less than 1.07 (the reference value for the cut-off point of Very Adequate), indicating that all of the categories were deemed very adequate by the experts consulted to form part of the App’s informative content on informal caregivers of stroke patients.

In addition, the responses’ means and standard deviations, as well as the coefficient of variation, were computed. As summarized in Table 5, the averages obtained from the first round of responses are greater than 4.5 out of five. On the other hand, the coefficient of variation for each category is less than 0.5, presenting an agreement among the participants.

In addition to the quantitative part, participants provided suggestions for implementing and improving each category (Table 6). As a result, the majority of the recommendations were aimed at improving content writing. Nevertheless, it was also suggested that infographics and explanatory videos be introduced to support the content, making the information more practical and accessible to informal caregivers. For this reason, despite the great consensus in this first round, suggestions and improvements to the content were implemented, and the second consultation round was held.

In the second round of consultation, the content with the improvements made (Table 6) and the statistical data obtained from the first round were sent for observation. As a result, responses indicated that 100% of respondents agreed with the scores and the improvements that were made to the content developed for the various categories.

The first round resulted in an agreement on the appropriateness and relevance of the content developed for informal caregivers of stroke patients, which will be hosted on the App for this population. Moreover, the suggestions made in this first round (mostly on the wording and the addition of graphic material) were used to improve the informative content. With the second round, it was discovered that the participants agreed with the scores and improvements recommended by their peers in the previous round.

## 4. Discussion

The advancement of technology has clearly been a driving force in providing remote health care, and smartphones have contributed to the development of e-Health, supporting health education and promotion (Guimaraes Marcelino et al., 2013) [18].

International literature refers to non-pharmacological psychosocial or psychoeducational strategies and interventions using telephony to support informal caregivers in their process (Romero Guevara et al., 2011; Corry et al., 2019) [19,20]. Furthermore, there are studies that display users’ preferences for receiving assistance and interventions through these devices, as well as their satisfaction after receiving it (Beaver et al., 2011) [21].

According to the literature, it can be concluded that the users’ experiences with e-Health interventions, such as informal caregivers, have been beneficial. Since it has been applied to provide health education, support decision-making, or foster the learning of new skills (Beaver et al., 2011; Chi and Demiris, 2015) [21,22].

As a result, the Delphi technique was used to fine-tune the content, which was based on manuals and clinical practice guidelines, in order to create informative content for informal caregivers of stroke patients to be included in an App aimed at improving the life quality of this population segment.

One of the most frequently discussed issues of using a health app is the reliability and accuracy of the information it contains. A review of 12 tertiary prevention mobile apps declared that the collaboration of professional experts in the design and development improves the information quality (Martínez Moreno et al., 2015) [23].

According to the WHO Bellagio eHealth Evaluation Group, rigorous eHealth evaluation is required to generate evidence and promote the integration and appropriate use of validated and accredited technology with quality, evidence-based information to improve health and reduce health inequalities (WHO|WHO Guideline, s. f.) [24].

Therefore, we were able to obtain the consensus of different experts in the field, all of whom had a medium-high level of competence by employing the Delphi technique. Furthermore, the content’s reliability was improved, with it being rated as very appropriate for the objectives set, as Boudreaux et al. pointed out (Boudreaux et al., 2014) [25].

According to the participating experts, the content offered should emphasize the wording of the development, making the information more approachable, practical, and positive for the target population. On the other hand, they believed that the inclusion of graphical elements and videos that aid in synthesizing and clarifying key concepts was essential. These aspects are in line with what a systematic review identified as providing greater satisfaction for users of health apps (Crow et al., 2002) [26].

Finally, there was complete agreement on the relevance of including the social support and resources available to them at regional and national levels. To that end, they proposed that it be improved to include and highlight the work of social workers as an important pillar, to which they could turn to.

### Limitations

A large amount of information on stroke, as well as the scarcity of data on caregivers’ roles and needs, could be assumed as the study’s main limitations. As few studies have figured out the problems and concerns of stroke caregivers, this aspect will be assessed through qualitative research in the App’s piloting.

In addition, another important aspect to highlight as a limitation of the study is the fact that no caregivers, patients or patient representatives were included in the expert group. This approach will be addressed in the piloting and validation of the App, so that real-time information will be improved.

## 5. Conclusions

It can be concluded that the content developed by the research group and agreed upon by experts using the Delphi technique could be used as part of a health App designed to improve the quality of life of informal caregivers of stroke patients. This assertion is supported by the results obtained in the different rounds of expert consultation, which allowed a high degree of consensus to be reached among the experts.

## Figures and Tables

**Table 1:** The content prepared by the researchers and offered to the experts in the first round.

Categories	Content
**Disease (stroke)**	In this first category, they are given detailed information about stroke, how to recognize it, prevention and risk factors, aftereffects, rehabilitation, and the activities that the nurse will be limited from doing after it.
**Caregivers**	The caregivers’ category discusses the concept of informal caregiving as well as the changes and consequences of taking on such a role.
**Tips**	There is a decalogue of tips for caregivers and an explanation that reinforces these tips in this class: asking for help, taking care of themselves, and learning new communication skills.
**Resources**	The term “Resources” refers to the different types of social resources available to informal caregivers and the patients they care for. These aids are intended to improve the population’s quality of life. Dependency law, Tele-assistance, and residences are a few examples.
**Associations**	The associations’ section includes a list of national and local associations of informal caregivers. They are also asked to contribute new partnerships that are not on the list.
**Incorporating videos**	They are given a link to watch videos on mobilization techniques and physical exercise. We are also looking for fresh ideas.

**Table 2:** Socio-demographic data of participants.

Participant No.	Sex	Education	Occupation	Years of Experience
**1**	Female	Degree in Nursing	Inpatient neurology nurse	15
**2**	Male	Degree in Nursing	Inpatient neurology nurse	>5
**3**	Female	Degree in Nursing	Inpatient neurology nurse	8
**4**	Female	Degree in Nursing	Inpatient neurology nurse	>5
**5**	Female	Degree in Nursing	Inpatient neurology nurse	10
**6**	Male	Degree in Nursing and Graduate in Psychology	Primary Care Nurse	16
**7**	Female	Degree in Nursing	Inpatient neurology nurse	12
**8**	Female	Degree in Nursing	Nurse Case Manager	19
**9**	Female	Degree in Nursing	Nurse in critical care unit	10
**10**	Male	Degree in Social Education	Director General for Dependency Care	16
**11**	Female	Degree in Nursing	Sub-Directorate for Chronic Care and Socio-Sanitary Coordination	30
**12**	Female	Degree in Nursing and Graduate in Economics	Sub-Directorate for Chronic Care and Socio-Sanitary Coordination	21
**13**	Female	Degree in Medicine	Inpatient neurologist	15

**Table 3:** Experts’ level of competence.

Experts	Kc *	Ka **	K ***	Level of Competence
**1**	0.8	0.5	0.65	Medium
**2**	0.7	0.5	0.6	Medium
**3**	0.6	0.5	0.55	Medium
**4**	0.7	0.8	0.75	Medium
**5**	0.5	0.5	0.5	Medium
**6**	0.5	0.5	0.5	Medium
**7**	0.7	0.8	0.75	Medium
**8**	0.6	0.8	0.7	Medium
**9**	0.7	0.8	0.75	Medium
**10**	0.9	0.8	0.8	High
**11**	0.8	0.7	0.75	Medium
**12**	0.7	0.8	0.75	Medium
**13**	0.9	0.8	0.85	High

* Kc: coefficient of knowledge; ** Ka: argumentation coefficient; and *** K: coefficient of competence.

**Table 4:** Results obtained for the validation of the information by the Expert Consultation method (first round).

Aspects	Very Adequate	Quite Adequate	Adequate	Not Adequate	Sum	Average	N-P
Stroke	1.02	1.02	3.09	3.09	8.22	2.055	−0.3
Caregivers	0.73	1.02	3.09	3.09	7.93	1.9825	−0.23
Tips	0.923	1.42	3.09	3.09	8.523	2.1307	−0.38
Resources	1.42	1.02	1.42	3.09	6.95	1.7375	0.01
Associations	0.923	3.09	3.09	3.09	10.193	2.5482	−0.798
Videos	1.42	3.09	3.09	3.09	10.69	2.6725	−0.92
Sum	6.436	10.66	16.87	18.54	52.506		
Cut-off points	1.07	1.77	2.81	3.09		N = 1.7502	

**Table 5:** Statistical results of the first round.

Aspects	Mean	Standard Deviation	Coefficient of Variation
**Stroke**	4.69	0.75	0.16
**Caregivers**	4.61	0.77	0.16
**Tips**	4.85	0.55	0.11
**Resources**	4.61	0.96	0.2
**Associations**	4.92	0.27	0.05
**Videos**	4.84	0.37	0.07

**Table 6:** Suggestions and improvements made to the content after the first round of consultation.

Categories	Suggestions from Participants	Improvements Made after the First Round
**Stroke**	Improve the information wording: use more accessible language, language that encourages patient autonomy, and less technical language. Summarize the content in key points. Add graphic elements, infographics, images, and so on to the information.	The language used to create the content was improved to make it more understandable, practical, and approachable. Infographics were incorporated to summarize the most important points of the content.
**Caregivers**	Enhance wording to make the content more understandable in some parts, write it positively, and bring it closer to the person receiving it (in this case, the caregiver). Introduce infographics to support the text.	The wording was enhanced to make the language more accessible, as well as the wording of some difficult-to-understand sections. Infographics were included to supplement the information developed.
**Tips**	Improve the writing grammar and write less formally. Apply images and infographics to support the content, as in previous examples.	Content writing was improved. Infographics were introduced to support the information developed.
**Resources**	Mention social workers and ask for assistance.	The assistance and the places where they can go for more information and support (social workers) were specified.
**Associations**	Include official organizations, national and community associations, and help pages in the resolution of bureaucracy. Provide association names that could be added.	The associations provided in the suggestions were evaluated and located in this category. The contact and bureaucracy resolution help pages were placed in the resources category as they are more relevant to the content.
**Videos**	Animate videos on all content. Provide videos for the self-care caregiver (stretching, strengthening, etc.). Present breathing and relaxation techniques. Prepare videos to help the patient with daily tasks.	Animated videos were created to explain key points of the content as well as the caregiver’s maintenance.

## Data Availability

Not applicable.
